# Workforce requirements for comprehensive ischaemic stroke care in a developing country: the case of Saudi Arabia

**DOI:** 10.1186/s12960-019-0408-y

**Published:** 2019-12-02

**Authors:** Fahmi Al-Senani, Mohammad Salawati, Mohammed AlJohani, Matthieu Cuche, Valeska Seguel Ravest, Simon Eggington

**Affiliations:** 1 Department of Neurology, National Neurosciences Institute, King Fahad Medical City, Riyadh, Saudi Arabia; 2 Neurovascular Health Economics and Reimbursement, Medtronic International Trading Sàrl, Tolochenaz, Switzerland; 3 Neurovascular Health Economics and Reimbursement, Medtronic Ltd, Watford, UK; 4 Corporate Health Economics and Reimbursement, Medtronic International Trading Sàrl, Tolochenaz, Switzerland; 5 Medtronic International Trading Sàrl, Route du Molliau 31, 1131, Tolochenaz, Switzerland

**Keywords:** Workforce planning, Ischaemic stroke, Saudi Arabia, Stroke epidemiology

## Abstract

**Background:**

Ischaemic stroke care requires a co-ordinated multi-disciplinary approach to optimise patient outcomes. Current care provision in Saudi Arabia is below international recommendations, and with increasing patient numbers, variable access to new therapies, and sub-optimal co-ordination of staff, the Kingdom’s Ministry of Health has prioritised strategies to develop stroke care. Our objective was to use local epidemiological data to predict stroke incidence and to combine this with international staffing recommendations to estimate future staff requirements and their costs over a 10-year period.

**Methods:**

We researched existing stroke services and staff availability within Saudi Arabia to establish current provision, undertook epidemiological modelling to predict stroke incidence, and used international staffing recommendations for acute and rehabilitation services to develop a care pathway to provide state-of-the-art stroke services. This information was used to determine the additional staff requirements, and their costs, across the Kingdom.

**Results:**

Our research concluded that current staff numbers and services are inadequate to cope with the projected increase in the number of stroke cases. In order to provide acute and rehabilitation services which use the latest technologies, re-organisation of existing staff and services would be required, together with significant investment in new staff across several disciplines. An estimated additional 43.8 full-time equivalent stroke neurologists would be required, plus 53.5 full-time equivalent interventional neuroradiologists in addition to expansion of occupational therapy and psychology services. The total cost of additional staff over 10 years was estimated to be 862 390 778 Saudi Riyals ($229 970 874).

**Conclusions:**

Providing high-quality care for ischaemic stroke patients would involve significant investment in new staff in Saudi Arabia. Further research is required on the applicability of international staffing ratios to countries where there is a significant workforce gap. Nevertheless, this analysis provides a framework to inform stroke care planning and can be adapted to other regions or countries.

## Background

Stroke is a leading cause of mortality and morbidity around the world, with around 15 million strokes annually and 5.8 million deaths from stroke-related causes [1]. The main risk factor for stroke is age [2], with incidence rising sharply in the elderly and leading to concerns about the future burden of stroke in regions with growing and ageing populations. Stroke also represents a major economic burden, with acute and long-term care representing up to 5% of all healthcare spending in some regions [3, 4].

Stroke care service planning has therefore been a growing area of research, with studies undertaken in the United Kingdom and in Canada to formulate national guidelines and recommendations on workforce and infrastructure requirements for the provision of high-quality care with the goal of improving patient outcomes [5–7]. These guidelines recommend the use of dedicated hospital units to provide a multi-disciplinary approach to acute stroke care, comprising staff from disciplines including stroke neurology, occupational therapy, physiotherapy, and speech and language therapy. They also provide recommendations regarding the use of brain imaging to aid diagnosis, and the wider adoption of the most effective interventions according to stroke type and severity.

The Kingdom of Saudi Arabia has a young population, with almost 60% of the population under the age of 30 [8] and a significant number of non-Saudi residents who come to the country to work and who generally return to their country of origin upon retirement. The Saudi healthcare system is funded and provided largely by the Ministry of Health, supplemented by hospitals run by other government agencies and a growing private sector, with the majority of services found in the major cities [9]. Healthcare services are more patchy in rural areas, where population density is much lower. Despite significant improvements in the quality of healthcare services in recent decades, the Saudi Ministry of Health has identified a range of strategies to meet the demands of a growing and ageing population, but there remains a substantial shortfall of trained staff to meet these targets [10].

Recent statistics suggest that stroke is the Kingdom’s second-largest killer, being accountable for 14 400 deaths in 2012 [11], yet the country has only two specialist centres to provide high-quality care to stroke victims. A gap remains in the use of evidence-based interventions such as intra-venous tissue plasminogen activator (IV-tPA) for acute ischaemic strokes and mechanical thrombectomy for large-vessel occlusions, and in some of the varied staff types required to manage stroke patients. These factors have led the Ministry of Health to prioritise stroke care as a key component of its healthcare planning activities, including the optimisation of existing resources and workforce.

Our objective was to use local population and stroke incidence data to predict ischaemic stroke rates in Saudi Arabia, and to combine this information with existing staff availability within the Kingdom and with international workforce recommendations to estimate future staff requirements and costs over 10 years. The focus of the analysis was staff requirements for acute stroke care and inpatient rehabilitation in the immediate aftermath of stroke (outpatient and primary care services are a small proportion of the overall burden of stroke care and were thus excluded).

## Methods

### Overview of gap analysis

A wide body of evidence exists on methods for workforce supply estimation, covering both the supply element and the demand for services. Some such methods include regression-based models, simulation models, Markov chains, and system dynamics. Epidemiological modelling is commonly used to determine demand for health care services, considering population demographics, health status, and the types of service required [12, 13]. Existing supply has typically been estimated via national databases, online sources (such as the indicators available from the OECD), or tailored surveys in specific countries [12]. The future supply of healthcare professionals, meanwhile, is often modelled via a stock-and-flow approach, incorporating the training and employment of new healthcare specialists, retirement, immigration, and emigration, and taking into account practical changes such as flexible working hours and improvements in hospital efficiency [14]. Our approach used some elements of these methods (such as epidemiological modelling to determine future demand for stroke care services); however, we did not seek to model future staff supply via the methods described above. Instead, we combined the estimates of future demand with those of current staff availability and used international standards for stroke care as the basis for calculating the additional resources required.

A five-step process was followed to quantify the workforce gap for ischaemic stroke. First, comprehensive data collection was performed to determine the current workforce in the Kingdom for ischaemic stroke management and the existence of specialist stroke units. Data were collected for each region and supplemented with interviews with officials from the Saudi Ministry of Health. Second, an epidemiological model was created to predict population changes over the next decade, which was combined with local stroke incidence data to estimate the number of strokes occurring each year. Third, a model of future care was developed, reflecting changing patient care options and the availability of new therapies for specific groups of stroke patients, and based around three facility types. Fourth, a review of stroke care guidelines and recommendations from around the world was undertaken to inform the staff-to-patient ratio assumptions for the full range of specialists required within each of the three care facility types. Fifth, these elements were combined with future expectations of the use of novel stroke interventions to estimate staff requirements (and their costs) in stroke units and rehabilitation facilities over 10 years.

### Current stroke workforce in Saudi Arabia

An extensive search of multiple sources was undertaken to ascertain the current workforce for stroke care within Saudi Arabia. Workforce data were collected from the Ministry of Health’s statistical yearbook from 2016 [15]; this included all physicians by specialisation, nurses, and all allied healthcare staff, with the exception of dietitians and psychologists, by geographical region. The data collection was supplemented via a survey of all hospitals providing stroke services within Saudi Arabia and was co-ordinated by one of the authors (FA-S) as part of the Ministry of Health’s Stroke Vision 2030 programme. Regional directors in the Kingdom were contacted by email via the Ministry of Health, with the survey cascaded down to individual hospitals to determine staff provision dedicated to stroke care. Thus, hospital-level data on staff in each of the specialties was recorded and scaled up to the national level. The data collected were validated by the authors to confirm accuracy.

### Epidemiological modelling

To accurately determine staffing needs in the Kingdom over the next decade, robust estimates of stroke incidence were required to reflect population and demographic changes. Epidemiological modelling was therefore undertaken using local population, mortality, and stroke incidence data to predict the number of first strokes occurring each year over the next decade.

Data from the Saudi General Authority for Statistics were obtained to form the basis of the current population, split by age category and gender and available for both native Saudis and non-Saudis [8], allowing the populations to be modelled separately to reflect the effect of immigration and emigration. Mortality data were applied to the baseline population data to model deaths occurring over a 10-year period [8]. The movement of non-Saudis in and out of the country (many arrive to work but return to their home countries upon retirement) was also factored into the analysis. Thus, year-by-year population estimates were obtained by modelling births, deaths, and the gradual ageing of the population.

These data were then combined with Saudi age- and gender-specific rates of first stroke to estimate the number of first strokes occurring per year, of which 85% were assumed to be ischaemic [16, 17]. In each year, the population was stratified into age groups and multiplied by the stroke incidence rates for the corresponding age bands to estimate the number of new strokes occurring. The age- and gender-specific stroke risks were assumed to be constant over the horizon of the analysis, and therefore the growth and ageing of the underlying population drove the changes in predicted number of strokes over time. Recurrent strokes were excluded due to a lack of robust data on their incidence.

### Future stroke care structure and patient management

We then developed a generic structure for patient management, using the information obtained from the research and interviews described previously, and a framework was created according to expected future treatment approaches. In Saudi Arabia, only around 5% of stroke patients are admitted to acute stroke units located in comprehensive stroke centres with specialist staff. Thus, most patients do not have access to treatments such as intravenous tissue plasminogen activator (IV-tPA) and mechanical thrombectomy (MT). The proposed care model was based around increased adoption of IV-tPA and MT, drawing on international evidence regarding patient eligibility and applying a gradual uptake of these interventions [17–21].

Evidence suggests that around 85% of all strokes are ischaemic [22], and of these, 40% are large-vessel occlusions (60% are other-vessel occlusions) [17]. Amongst patients with LVO, the model assumes 5% use of MT in year 1 (either as a single intervention or in combination with IV-tPA), rising to 50% by year 10. All remaining LVO patients, plus all patients with OVO, were assumed to receive IV-tPA alone or non-thrombolytic therapy. Across all LVOs and OVOs, the model assumed that the proportion of patients treated with MT would increase from 2% in year 1 to 20% in year 10.

**Fig. 1:**
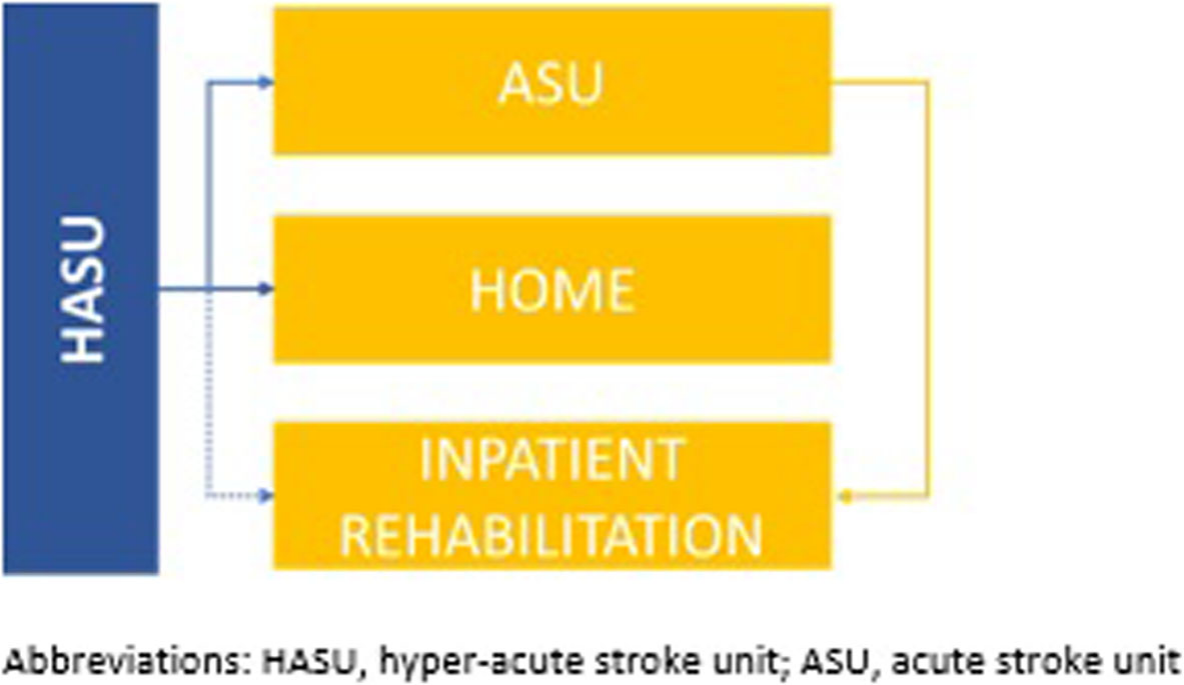
Proposed care model for Saudi Arabia

To reflect this shift in treatment mix, the model focused on three types of stroke facility, each requiring a different mix of specialist staff: hyper-acute stroke units (HASUs), acute stroke units (ASUs), and inpatient rehabilitation centres (Fig. 1). Under this care model, an increasing proportion of ischaemic stroke patients would be admitted to a HASU, regardless of stroke severity and the treatment to be administered, and would subsequently be discharged to one of the following locations: ASU, home, or inpatient rehabilitation. Our approach considered a phased introduction of HASUs, ASUs, and inpatient rehabilitation services over time, with 11% of patients assumed to be managed in a HASU in year 1, rising to 70% by year 10 with the establishment of new services. In the absence of sufficient capacity to allow all patients to be admitted to a HASU, all remaining patients were assumed to be managed using existing stroke care services.

Alternative models of care exist and depend on local resources and the feasibility of re-organising existing services. However, for the analyses presented here, we have adopted this approach to future stroke care. Under this model, we assumed that 50.5% of patients would be discharged directly to an ASU, 16.6% to inpatient rehabilitation (either directly from the HASU, as indicated by the dotted line in Fig. 1, or following a spell in the ASU), and 37.7% to home, based on interviews with stroke experts in Saudi Arabia.

### Review of practice guidelines and staffing ratios

The fourth phase of the study involved a review of international stroke care recommendations to identify best practices regarding workforce requirements for HASUs, ASUs, and inpatient rehabilitation. The review considered studies from countries with more developed and co-ordinated stroke care services and which had published detailed workforce requirements, and we adopted these guidelines as ‘gold-standard’ approaches for the Saudi healthcare system to adopt. The national stroke clinical guidelines issues by the Royal College of Physicians in the United Kingdom and recommendations from the Canadian Stroke Network were used as the basis for staffing ratios for care in HASUs and ASUs [5, 6]. Both documents provide detailed recommendations regarding the establishment of new stroke units and guidance on staff requirements. Requirements for stroke rehabilitation units were based upon standards from the Australasian Faculty of Rehabilitation Medicine [7], which provide staff-to-patient ratios for inpatient stroke rehabilitation care. Although implementation of such recommendations and guidelines in countries with less developed systems is not straightforward, this approach provides a ‘best-practice’ target for evaluating requirements in Saudi Arabia and reflects the Ministry of Health’s willingness to adopt successful strategies. Such an approach provided a framework upon which future staff requirements could be estimated.

A detailed breakdown of the recommended staff needs from these countries is provided in Additional files 1, 2, and 3.

### Estimation of workforce requirements in Saudi Arabia

The staffing ratios described were used as the baseline assumptions for requirements in Saudi Arabia. However, differences in the countries’ models of care, combined with the geographic spread of the population in Saudi Arabia, meant that adjustments were necessary to ensure feasibility. The international recommendations were therefore supplemented by consultations with local stroke experts (including radiologists and rehabilitation staff) to develop Saudi-specific staffing ratios. These discussions allowed us to derive local staff-to-patient ratios that covered the full range of staff, with the exception of nurses, whose resources were assumed to be re-organised and re-distributed according to the new care model.

We used the stroke incidence projections and data on population density to model the expected number of ischaemic strokes per year in each of the Kingdom’s 13 regions, allowing a more realistic assessment of local needs to be captured and scaled up to a national level. Staff requirements were then determined by calculating the expected number of beds needed in the three care settings. Bed requirements for HASUs and ASUs in each region were calculated based on an assumed occupancy of 50 cases per bed per year. For inpatient rehabilitation, bed needs were based on 16.6% of patients requiring inpatient rehabilitation after discharge from the HASU; length of stay in the rehabilitation unit was based on a mean of 25 days, using data from Australia [23, 24].

The number of interventional neuroradiologists (INRs) required for the HASUs in each region was calculated based on the number of ischaemic strokes and an acceptable number of LVO stroke cases per INR per year. Given the learning curve involved in the use of MT, we assumed that the number of procedures per year undertaken by each INR would increase from 15 in year 1 to 63 by year 10. One full-time equivalent (FTE) stroke neurologist was assumed to be required to cover five beds within a HASU, and based on discussions with a Saudi rehabilitation expert, the number of FTE physicians specialising in physical medicine and rehabilitation within HASUs and ASUs was assumed to be 0.084 per five beds (equivalent to 0.5 FTE per 30 beds).

The final staffing ratios (per five beds) applied for the model are shown in Table 1, covering the various specialist staff types needed in each care setting. All of the numbers given in the table were taken directly from the stroke guidelines issued in the United Kingdom and in Canada, the details of which have been previously published [5, 6]. Nursing staff are considered outside the scope of the study since sufficient numbers were assumed to be available via re-organisation of existing staff.

**Table 1:** Staffing ratios (full-time equivalents) per five beds, by unit type

Staff type	HASU	ASU	IPR
Stroke neurologist	1	–	–
Interventional neuroradiologist	*	–	–
Physical medicine and rehabilitation physician	0.084	0.084	0.2
Internist	–	0.5	–
Physical therapist	0.73	0.84	1 (0.75)
Occupational therapist	0.68	0.81	0.75
Speech and language therapist	0.34	0.4	0.75
Psychologist	0.2	0.2	0.2
Dietitian	0.15	0.15	0.25

*The ratio for INR is per LVO cases per year (1 INR per 50 cases per year). *HASU* hyper-acute stroke unit, *ASU* acute stroke unit, *IPR* inpatient rehabilitation

### Cost analysis

It was assumed that the new HASUs, ASUs, and rehabilitation units could be created within existing hospitals via reconfiguration of current infrastructure. Therefore, the economic analysis focused around the costs of new staff requirements. Salary data from the Saudi Ministry of Health were obtained for each staff type [25], to which 40% overheads were added (Additional file 4).

Using the staffing requirements described in the previous section, the total costs of the new care model were estimated by applying the staff unit costs to the individual resource components. In order to focus on the cost of staff provision, we have excluded costs related to the acquisition of new equipment (for example, bi-plane angiography suites).

## Results

Table 2 shows the current availability of specialist staff in Saudi Arabia, based on the research and interviews conducted. These are resources that are dedicated to stroke care, and do not cover other disease areas.

**Table 2:** Current stroke staff available in Saudi Arabia

Resource	Current availability (FTE)
Interventional neuroradiologist	21
Stroke neurologist	22
Physical medicine and rehabilitation physician	9.83
Physiotherapist	240
Occupational therapist	2.20
Speech and language therapist	1.4
Psychologist	0.74
Dietitian	49
Nurse	2663
Social worker	0.4

*FTE* full-time equivalent

The research also concluded that there is currently one HASU bed per 500 stroke cases, which is well below international recommendations and highlights the need for a broader HASU service.

Based on the input data and analytical approach described, the model predicted a 67% increase in first ischaemic stroke over the next 10 years, from 14 071 in year 1 to 23 482 in year 10. Based on the increasing use of mechanical thrombectomy (from 281 cases in year 1 to 4696 cases in year 10) and a phased introduction of the new pathway (in which 70% of cases are managed within a HASU by year 10), a total of 329 additional HASU beds are predicted to be required over the 10-year period. Year-by-year stroke projections, mechanical thrombectomies, and HASU bed requirements are given in Additional file 5.

Table 3 shows the predicted number of additional staff required in each field, based on increasing use of HASUs as the primary destination. In each case, the expected number of full-time equivalents is given, with total costs aggregated across all staff types and across all three service types. Service-specific results are provided in the Additional files 5, 6, and 7.

**Table 3:** Number of new full-time equivalents and associated total cost by year

Year	Stroke neurologist	Interventional neuroradiologist	Physical medicine and rehabilitation physician	Internist	Physiotherapist	Occupational therapist	Speech and language therapist	Psychologist	Dietitian	Social worker	Cost—Saudi Riyals (US dollars)
1	0	0	0	0	0	10.9	6.4	2.9	0	1.4	4 791 190 (1 277 651)
2	0	0	0	0	0	13.9	8.3	3.9	0	1.9	10 970 835 (2 925 556)
3	0	3.3	0	0	0	15.6	9.3	4.3	0	2.1	21 494 757 (5 731 935)
4	6.2	6.3	1.9	0	0	17.2	10.3	4.8	0	2.3	42 266 491 (11 271 064)
5	8.9	13.1	3.6	0	0	18.9	11.3	5.3	0	2.5	74 076 646 (19 753 772)
6	8.3	8.5	3.4	0	0	17.5	10.5	4.9	0	2.4	99 703 562 (26 587 617)
7	11.1	16.2	4.6	0	0	23.5	14.0	6.6	0	3.1	139 209 001 (37 122 400)
8	3.2	2.7	1.3	0	0	6.8	4.0	1.9	0	0.9	148 415 453 (39 577 454)
9	3.1	2.5	1.3	0	0	6.6	3.9	1.8	0	0.9	157 196 716 (41 919 124)
10	3.0	1.1	1.2	0	0	6.3	3.8	1.8	0	0.9	164 266 126 (43 804 300)
Total	43.8	53.5	17.4	0	0	137.1	81.9	38.2	0	18.3	862 390 778 (229 970 874)

The results in Table 3 indicate that a significant number of additional stroke specialists will be required across multiple disciplines to meet the expected increase in demand and the model of care. In particular, 43.8 FTE stroke neurologists and 53.5 FTE interventional neuroradiologists are predicted to be required to provide the expertise for managing patients in HASUs and ASUs. Furthermore, rehabilitation services would also require additional staff, including in the areas of physical medicine and rehabilitation (17.4 FTE), occupational therapy (137.1 FTE), speech and language therapy (81.9 FTE), and psychology (38.2 FTE). The total predicted costs of these staff are SR 862 390 778 ($229 970 874) over 10 years. Based on the survey of current staff availability and the proposed staffing requirements for Saudi Arabia, no further internists, physiotherapists, or dietitians would be required.

## Discussion

Stroke care has been identified as a key focus for the coming decade in Saudi Arabia, given the ageing population and inequity of care across the Kingdom. This study has presented a workforce gap analysis to quantify future staff requirements based around a care model for ischaemic stroke in which patients are managed via acute and hyper-acute stroke units and inpatient rehabilitation. We undertook a nationwide survey to establish the current workforce dedicated to stroke care and developed an epidemiological model to project the number of first ischaemic strokes over 10 years. Informed by international recommendations on staff-to-patient ratios for the different care locations and based on discussions with local stroke experts, we proposed a care model to determine staff requirements in each setting, scaling up local-level projections to the national level. These data were then combined with staff salary information to estimate the workforce costs associated with this service expansion.

Based on this approach, our results indicate that significant expansion of existing hyper-acute stroke units would be needed to meet rising demand, with 329 additional beds required over the 10-year period to manage patients according to the proposed care model. From our analysis of existing services, however, these additional beds could be found via reconfiguration of existing services and were beyond the scope of the study due to our stated focus on staff requirements. The most substantial areas of development are expected to be the training and hiring of additional stroke neurologists (43.8 FTE) and interventional neuroradiologists (53.5 FTE) to meet the expanded use of mechanical thrombectomy and the increasing proportion of patients initially managed within a HASU. Significant investment in rehabilitation staff such as occupational therapists, psychologists, and speech and language therapists would also be required. Staff disciplines in which sufficient resources are already in place include physiotherapy and dietetics. Total staff costs over 10 years were estimated at SR 862 390 778 ($229 970 874).

There are some limitations to our study. First, our staff projections were based on international recommendations for the different care settings and thus may not be directly transferable to Saudi Arabia. We selected staff ratios from countries with highly developed services to provide a ‘gold-standard’ target for Saudi Arabia. However, a variety of other care configurations (and staff ratios) exist, and further research is required to understand the applicability of these strategies to countries developing their stroke services. In the absence of a comprehensive stroke care service in Saudi Arabia, we believe that using such international benchmarks as the basis for future requirements represents the most appropriate scenario to consider. We used regional population data to localise the future staff requirements, and subsequently scaled up to the national level. Nevertheless, provision of services in sparsely populated areas is complex, and specific strategies may be needed to provide equitable care across the Kingdom. For this reason, we revised the recommended staffing ratios based on interviews with stroke experts in Saudi Arabia to ensure greater applicability.

Second, our analysis is built around epidemiological modelling to predict the number of ischaemic strokes. We used data from the 1990s to inform stroke rates in Saudi Arabia despite more recent data having been published [26]. The latter data were not used due to concerns about the low stroke rate observed amongst elderly females, a pattern inconsistent with data from other studies [27–29]. The exclusion of recurrent strokes may mean that our projections underestimate staff requirements in the future; however, studies have shown that up to 40% of stroke patients do not receive hospital treatment [30], which is particularly common amongst elderly patients [31, 32], and this may offset the exclusion of recurrent stroke. Extrapolation of the population and stroke incidence data mean that the estimates of stroke burden over 10 years are subject to uncertainty, and thus, the projected staffing requirements should also be interpreted with this uncertainty in mind. Use of alternative stroke incidence data would lead to different estimates of staff requirements; nevertheless, the population growth projection of 12.8% over 10 years is consistent with estimates of 2% per year from the World Bank [33]. Hence, this growing and ageing population would lead to significant increases in the number of strokes regardless of the underlying data used to represent stroke incidence.

Third, our economic analysis focused on the salary costs of the additional staff needed to meet the increasing demand, patient pathways, and adoption of novel technologies. We therefore excluded any infrastructure and equipment costs and the training and attrition of staff over time.

It is important to note the distinction between headcounts and FTEs as measures of staff numbers. A headcount approach considers each person once, regardless of the number of hours worked (for example part-time versus full-time workers), whereas an FTE approach is based on the number of hours worked expressed as a proportion of the normal hours of a full-time employee. This has the effect of making part-time and full-time workers comparable. The FTE is a useful measure to support the allocation of employees across different departments based on the required work levels and any budget constraints. The FTE therefore appears the most appropriate measure to use for workforce planning in this study since it focuses specifically on ischaemic stroke patients.

Stroke care co-ordination is a complex issue, requiring specialist care across a wide range of disciplines. In Saudi Arabia, a shortage of trained staff has been highlighted, yet part of the solution identified involves improved organisation and co-ordination of the existing workforce. Sub-optimal patient outcomes can in part be attributed to such organisational factors. For example, many hospitals do not have a HASU or ASU but admit stroke patients, and thus, an absence of specialist staff and equipment impacts patient outcomes. Similarly, some hospitals have stroke consultants but no HASU or ASU in which to provide high-quality care, and such consultants may be underused as a result. Thirdly, not all patients are admitted to hospital and thus do not receive appropriate treatment. Improvement of stroke care provision must therefore address not only the development of new services and the hiring of new staff, but the re-organisation of existing services and workforce. These principles extend to any country wishing to improve the level of stroke care, ensuring more efficient use of staff resources, greater use of the latest technologies, and improved patient outcomes. Local data on current staff provisions, organisation of care, stroke incidence, mortality, and cost could be applied to adapt the analysis to other countries.

## Conclusions

This study provides a framework for estimating staff requirements associated with a transition to more advanced care of ischaemic stroke patients, using local epidemiology data together with international recommendations on acute and rehabilitation services. The model proposed here can be adapted to other countries wishing to develop stroke service provision in the future and can be used at a national or regional level to inform staff planning and to explore ways of ensuring geographical coverage of stroke services. This detailed approach is necessary due to the multi-disciplinary nature of stroke care, but could be applied more broadly in other disease areas to quantify workforce requirements and particularly to conditions of the elderly and where ageing populations are likely to increase the burden on healthcare services. Careful consideration of the translation of staffing ratios from one country to another is important to ensure applicability to the target country.

## Supplementary information

**Additional file 1:** United Kingdom clinical guidelines on minimum staffing levels for hyper-acute stroke units and acute stroke units. Full-time equivalents recommended in hyper-acute and acute stroke units.

**Additional file 2:** Canadian recommendations on staffing levels for acute stroke units and inpatient rehabilitation. Full-time equivalents recommended for acute stroke units and inpatient rehabilitation services.

**Additional file 3:** Australian recommendations on staff-to-patient ratios for inpatient rehabilitation services. Full-time equivalents recommended for each staff type for inpatient rehabilitation.

**Additional file 4:** Unit costs used in the model. Summary of the unit cost of each staff type included in the analysis.

**Additional file 5:** Hyper-acute stroke units - number of new full-time equivalents and associated cost by year. Year-by-year results of staff requirements in hyper-acute stroke units and estimated total cost.

**Additional file 6:** Acute stroke units - number of new full-time equivalents and associated cost by year. Year-by-year results of staff requirements in acute stroke units and estimated total cost.

**Additional file 7:** Inpatient rehabilitation - number of new full-time equivalents and associated cost by year. Year-by-year results of staff requirements for inpatient rehabilitation services and estimated total cost.

## Data Availability

The datasets used and analysed are available from the corresponding author upon reasonable request.

## Abbreviations

ASU: Acute stroke unit
CT: Computed tomography
FTE: Full-time equivalent
HASU: Hyper-acute stroke unit
INR: Interventional neuroradiologist
IPR: Inpatient rehabilitation
IV-tPA: Intra-venous tissue plasminogen activator
LVO: Large-vessel occlusion
MRI: Magnetic resonance imaging
MT: Mechanical thrombectomy
OTA: Occupational therapy assistant
OVO: Other vessel occlusion
PTA: Physiotherapy assistant
RN: Registered nurse
SR: Saudi Riyals
USD: United States dollars
