# Supplementary material for: Workforce requirements for comprehensive ischaemic stroke care in a developing country: the case of Saudi Arabia
Source: Hum Resour Health. 2019 Dec 2;17:90. doi: 10.1186/s12960-019-0408-y (PMC6889528; doi:10.1186/s12960-019-0408-y)
Supplement: Supplementary file 1 — Additional file 1: United Kingdom clinical guidelines on minimum staffing levels for hyper-acute stroke units and acute stroke units. Full-time equivalents recommended in hyper-acute and acute stroke units. [file 12960_2019_408_MOESM1_ESM.docx]

**Additional file 1.** *United Kingdom clinical guidelines on minimum staffing levels for hyper-acute stroke units and acute stroke units*

| Unit Type | Physio-therapist | Occupational Therapist | Speech & Language Therapist | Psychologist | Dietitian | Nurse | Stroke Consultant |
| --- | --- | --- | --- | --- | --- | --- | --- |
|  | Full-Time Equivalent per 5 Beds | | | | | FTE per Bed |  |
| HASU | 0.73 | 0.68 | 0.34 | 0.20 | 0.15 | 2.9 | 6 |
| ASU | 0.84 | 0.81 | 0.40 | 0.20 | 0.15 | 1.35 | 6 |

Based on a 24/7 service. Abbreviations: HASU, hyper-acute stroke unit; ASU, acute stroke unit; FTE, full-time equivalent
Source: National Clinical Guideline for Stroke. Royal College of Physicians, UK, 2016.
