# Supplementary material for: Workforce requirements for comprehensive ischaemic stroke care in a developing country: the case of Saudi Arabia
Source: Hum Resour Health. 2019 Dec 2;17:90. doi: 10.1186/s12960-019-0408-y (PMC6889528; doi:10.1186/s12960-019-0408-y)
Supplement: Supplementary file 2 — Additional file 2: Canadian recommendations on staffing levels for acute stroke units and inpatient rehabilitation. Full-time equivalents recommended for acute stroke units and inpatient rehabilitation services. [file 12960_2019_408_MOESM2_ESM.docx]

**Additional file 2.** *Canadian recommendations on staffing levels for acute stroke units and inpatient rehabilitation*

| Discipline | Full-Time Equivalents |
| --- | --- |
| Nursing RN | 21 (acute), with 1 RN covering rehab 24/7 |
| Nursing RPN | 12 (rehab) |
| Physiotherapy | 2 (1 acute, 1 rehab) |
| Occupational therapy | 2 (1 acute, 1 rehab) |
| OTA/PTA | 2 (1 acute, 1 rehab) |
| Speech & language pathologist | 1.5 (acute & rehab) |
| Social work | 1.5 (acute & rehab) |
| Dietitian | 0.6 (acute) |
| Dietetic assistant | 0.4 (rehab) |
| Pharmacy | 0.5 (acute & rehab) |
| Pharmacy tech | 0.5 (acute & rehab) |
| Neuropsychology | 0.5 (rehab) |
| MD model (monthly coverage schedule) | Acute – 2 stroke neurologists & 2 internists  Rehab – 2 stroke neurologists |

Abbreviations: RN, registered nurse; OTA, occupational therapy assistant; PTA, physiotherapist assistant; MD, medical doctor
Source: A guide to the implementation of stroke unit care. Canadian Stroke Network, 2009.
