# Supplementary material for: Workforce requirements for comprehensive ischaemic stroke care in a developing country: the case of Saudi Arabia
Source: Hum Resour Health. 2019 Dec 2;17:90. doi: 10.1186/s12960-019-0408-y (PMC6889528; doi:10.1186/s12960-019-0408-y)
Supplement: Supplementary file 3 — Additional file 3: Australian recommendations on staff-to-patient ratios for inpatient rehabilitation services. Full-time equivalents recommended for each staff type for inpatient rehabilitation. [file 12960_2019_408_MOESM3_ESM.docx]

**Additional file 3.** *Australian recommendations on staff-to-patient ratios* for inpatient rehabilitation*

| Occupational Therapist | Physiotherapist | Health Assistant | Speech and Language Therapist | Clinical Psychologist | Neuro-Psychologist | Podiatrist | Dietitian | Social Worker | Exercise Physiologist |
| --- | --- | --- | --- | --- | --- | --- | --- | --- | --- |
| 1.5 | 1.5 | 0.5 | 1.5 | 0.2 | 0.6 | 0.2 | 0.5 | 1.0 | 0.5 |

* Ratios are per 10 inpatients
Source: Standards for the provision of inpatient adult rehabilitation medicine services in public and private hospitals. Australasian Faculty of Rehabilitation Medicine, 2011.
