# Supplementary material for: Workforce requirements for comprehensive ischaemic stroke care in a developing country: the case of Saudi Arabia
Source: Hum Resour Health. 2019 Dec 2;17:90. doi: 10.1186/s12960-019-0408-y (PMC6889528; doi:10.1186/s12960-019-0408-y)
Supplement: Supplementary file 4 — Additional file 4: Unit costs used in the model. Summary of the unit cost of each staff type included in the analysis. [file 12960_2019_408_MOESM4_ESM.docx]

**Additional file 4.** *Unit costs applied in the model*

| Staff Type | Annual Salary, SR (USD) * |
| --- | --- |
| Interventional neuroradiologist | 1 092 000 (291 200) |
| Stroke neurologist | 924 000 (246 400) |
| Physical medicine and rehabilitation physician | 252 000 (67 200) |
| Internist | 420 000 (112 000) |
| Physical therapist | 252 000 (67 200) |
| Occupational therapist | 201 600 (53 760) |
| Speech and language therapist | 252 000 (67 200) |
| Psychologist | 252 000 (67 200) |
| Dietitian | 201 600 (53 760) |
| Stroke nurse | 84 000 (22 400) |

* Including 40% overheads. Abbreviations: SR, Saudi Riyals; USD, United States Dollars
