# Supplementary material for: Workforce requirements for comprehensive ischaemic stroke care in a developing country: the case of Saudi Arabia
Source: Hum Resour Health. 2019 Dec 2;17:90. doi: 10.1186/s12960-019-0408-y (PMC6889528; doi:10.1186/s12960-019-0408-y)
Supplement: Supplementary file 5 — Additional file 5: Hyper-acute stroke units - number of new full-time equivalents and associated cost by year. Year-by-year results of staff requirements in hyper-acute stroke units and estimated total cost. [file 12960_2019_408_MOESM5_ESM.docx]

**Additional file 5.** *Hyper-acute stroke units - number of new full-time equivalents and associated cost by year*

| Year | Stroke Consultant | | Interventional Neuroradiologist | | Physical Medicine and Rehabilitation Physician | | Occupational Therapist | | Speech & Language Therapist | | Psychologist | | Cost per Year – Saudi Riyals (US Dollars) |
| --- | --- | --- | --- | --- | --- | --- | --- | --- | --- | --- | --- | --- | --- |
|  | FTE | Cost SR (USD) | FTE | Cost SR (USD) | FTE | Cost SR (USD) | FTE | Cost SR (USD) | FTE | Cost SR (USD) | FTE | Cost SR (USD) |  |
| 1 | 0 | 0 (0) | 0 | 0 (0) | 0 | 0 (0) | 3.51 | 707 563  (188 683) | 1.70 | 429 627  (114 567) | 0.94 | 236 416  (63 044) | 1 373 605 (366 295) |
| 2 | 0 | 0 (0) | 0 | 0 (0) | 0 | 0 (0) | 4.45 | 1 605 679 (428 181) | 2.23 | 990 949  (264 253) | 1.31 | 566 606  (151 095) | 3 163 234  (843 529) |
| 3 | 0 | 0 (0) | 3.30 | 3 598 532 (959 609) | 0 | 0 (0) | 4.99 | 2 612 178 (696 581) | 2.50 | 1 620 011 (432 003) | 1.47 | 936 642  (249 771) | 8 767 363  (2 337 963) |
| 4 | 6.22 | 5 743 463  (1 531 590) | 6.29 | 10 468 249  (2 791 533) | 0.52 | 131 373  (35 033) | 5.53 | 3 726 937 (993 850) | 2.76 | 2 316 736 (617 796) | 1.63 | 1 346 480 (359 061) | 23 733 238  (6 328 863) |
| 5 | 8.90 | 13 965 257  (3 724 069) | 13.08 | 24 748 465  (6 599 591) | 0.74 | 318 232  (84 862) | 6.05 | 4 946 752  (1 319 134) | 3.03 | 3 079 120 (821 099) | 1.78 | 1 794 941 (478 651) | 48 852 768  (13 027 405) |
| 6 | 8.28 | 21 617 005  (5 764 535) | 8.51 | 34 046 585  (9 079 089) | 0.69 | 492 136  (131 236) | 5.63 | 6 081 993  (1 621 865) | 2.82 | 3 788 646  (1 010 306) | 1.66 | 2 212 309 (589 949) | 68 238 674  (18 196 980) |
| 7 | 11.07 | 31 847 757 (8 492 735) | 16.18 | 51 719 644 (13 791 905) | 0.92 | 724 653  (193 241) | 7.53 | 7 599 865  (2 026 631) | 3.76 | 4 737 316  (1 263 284) | 2.21 | 2 770 350  (2 931 335) | 99 399 584  (26 506 556) |
| 8 | 3.19 | 34 799 148  (9 279 773) | 2.66 | 54 627 840 (14 567 424) | 0.27 | 791 730  (211 128) | 2.17 | 8 037 744  (2 143 398) | 1.09 | 5 010 990  (1 336 264) | 0.64 | 2 931 335 (781 689) | 106 198 787  (28 319 677) |
| 9 | 3.09 | 37 656 778 (10 041 807) | 2.46 | 57 310 933 (15 282 915) | 0.26 | 856 676  (228 447) | 2.10 | 8 461 712  (2 256 457) | 1.05 | 5 275 970  (1 406 925) | 0.62 | 3 087 206 (823 255) | 112 649 276  (30 039 807) |
| 10 | 3.00 | 40 425 059 (10 780 016) | 1.06 | 58 472 842 (15 592 758) | 0.25 | 919 591  (245 224) | 2.04 | 8 872 425  (2 365 980) | 1.02 | 5 532 666  (1 475 377) | 0.60 | 3 238 203 (863 521) | 117 460 786  (31 322 876) |
| Total | 43.75 | 186 054 467 (49 614 524) | 53.55 | 294 993 089 (78 664 824) | 3.65 | 4 234 391  (1 129 171) | 44.01 | 52 652 849 (14 040 760) | 21.96 | 32 782 031  (8 741 875) | 12.85 | 19 120 489  (5 098 797) | 589 837 315  (157 289 951) |

Abbreviations: FTE, full-time equivalent; SR, Saudi Riyals; USD, United States Dollars
