# Supplementary material for: Workforce requirements for comprehensive ischaemic stroke care in a developing country: the case of Saudi Arabia
Source: Hum Resour Health. 2019 Dec 2;17:90. doi: 10.1186/s12960-019-0408-y (PMC6889528; doi:10.1186/s12960-019-0408-y)
Supplement: Supplementary file 6 — Additional file 6: Acute stroke units - number of new full-time equivalents and associated cost by year. Year-by-year results of staff requirements in acute stroke units and estimated total cost. [file 12960_2019_408_MOESM6_ESM.docx]

**Additional file 6.** *Acute stroke units - number of new full-time equivalents and associated cost by year*

| Year | Physical Medicine and Rehabilitation Physician | | Occupational Therapist | | Speech & Language Therapist | | Psychologist | | Cost per Year – Saudi Riyals (US Dollars) |
| --- | --- | --- | --- | --- | --- | --- | --- | --- | --- |
|  | FTE | Cost SR (USD) | FTE | Cost SR (USD) | FTE | Cost SR (USD) | FTE | Cost SR (USD) |  |
| 1 | 0 | 0 (0) | 5.27 | 1 062 064 (283 217) | 2.60 | 654 039  (174 411) | 1.21 | 304 340  (81 157) | 2 020 443  (538 785) |
| 2 | 0 | 0 (0) | 6.63 | 2 399 332 (639 822) | 3.28 | 1 479 514  (394 537) | 1.64 | 717 077  (191 221) | 4 595 923  (1 225 580) |
| 3 | 0 | 0 (0) | 7.43 | 3 897 979  (1 039 461) | 3.67 | 2 404 605  (641 228) | 1.84 | 1 179 622  (314 566) | 7 482 206  (1 995 255) |
| 4 | 0 | 0 (0) | 8.23 | 5 557 823  (1 482 086) | 4.07 | 3 429 200  (914 453) | 2.03 | 1 691 920  (451 179) | 10 678 943  (2 847 718) |
| 5 | 0.87 | 218 240  (58 197) | 9.01 | 7 374 092 (1,966425) | 4.45 | 4 550 353  (1 213 428) | 2.22 | 2 252 497  (600 666) | 14 395 182  (3 838 715) |
| 6 | 0.86 | 435 619  (116 165) | 8.38 | 9 064 433  (2 417 182) | 4.14 | 5 593 773  (1 491 673) | 2.07 | 2 774 207  (739 788) | 17 868 032  (4 764 809) |
| 7 | 1.15 | 726 266  (193 671) | 11.21 | 11 324 499  (3 019 866) | 5.54 | 6 988 876  (1 863 700) | 2.77 | 3 471 758  (925 802) | 22 511 399  (6 003 040) |
| 8 | 0.33 | 810 112  (216 030) | 3.23 | 11 976 488  (3 193 730) | 1.60 | 7 391 338  (1 971 024) | 0.80 | 3 672 989  (979 464) | 23 850 928  (6 360 247) |
| 9 | 0.32 | 891 295  (237 679) | 3.13 | 12 607 765  (3 362 071) | 1.55 | 7 781 015  (2 074 937) | 0.77 | 3 867 828  (1 031 421) | 25 147 902  (6 706 107) |
| 10 | 0.31 | 969 939  (258 650) | 3.03 | 13 219 303  (3 525 148) | 1.50 | 8 158 508  (2 175 602) | 0.75 | 4 056 574  (1 081 753) | 26 404 324  (7 041 153) |
| Total | 3.85 | 4 051 472  (1 080 392) | 65.57 | 78 483 779  (3 525 148) | 32.38 | 48 431 222  (12 914 992) | 16.10 | 23 988 811  (6 397 016) | 154 955 283  (41 321 409) |

Abbreviations: FTE, full-time equivalent; SR, Saudi Riyals; USD, United States Dollars
