# Supplementary material for: Workforce requirements for comprehensive ischaemic stroke care in a developing country: the case of Saudi Arabia
Source: Hum Resour Health. 2019 Dec 2;17:90. doi: 10.1186/s12960-019-0408-y (PMC6889528; doi:10.1186/s12960-019-0408-y)
Supplement: Supplementary file 7 — Additional file 7: Inpatient rehabilitation - number of new full-time equivalents and associated cost by year. Year-by-year results of staff requirements for inpatient rehabilitation services and estimated total cost. [file 12960_2019_408_MOESM7_ESM.docx]

**Additional file 7.** *Inpatient rehabilitation - number of new full-time equivalents and associated cost by year*

| Year | Physical Medicine and Rehabilitation Physician | | Occupational Therapist | | Speech & Language Therapist | | Psychologist | | Social Worker | | Cost per Year – Saudi Riyals (US Dollars) |
| --- | --- | --- | --- | --- | --- | --- | --- | --- | --- | --- | --- |
|  | FTE | Cost SR (USD) | FTE | Cost SR (USD) | FTE | Cost SR (USD) | FTE | Cost SR (USD) | FTE | Cost SR (USD) |  |
| 1 | 0 | 0 (0) | 2.14 | 431 336  (115 023) | 2.14 | 539 171  (143 779) | 0.78 | 196 524  (52 406) | 1.37 | 230 111  (61 363) | 1 397 142  (372 571) |
| 2 | 0 | 0 (0) | 2.79 | 994 468  (265 192) | 2.79 | 1 243 085  (331 489) | 0.93 | 431 162  (114 976) | 1.86 | 542 962  (144 790) | 3 211 678  (856 447) |
| 3 | 0 | 0 (0) | 3.13 | 1 625 558  (433 482) | 3.13 | 2 031 947  (541 853) | 1.04 | 694 116  (185 098) | 2.09 | 893 568  (238 285) | 5 245 188  (1 398 717) |
| 4 | 1.42 | 356 885  (95 169) | 3.47 | 2 324 528  (619 874) | 3.47 | 2 905 660  (774 843) | 1.16 | 985 353  (262 761) | 2.31 | 1 281 884  (341 836) | 7 854 311  (2 094 483) |
| 5 | 2.02 | 866 780  (231 141) | 3.79 | 3 089 370  (823 832) | 3.79 | 3 861 712  (1 029 790) | 1.26 | 1 304 037  (347 743) | 2.53 | 1 706 797  (455 146) | 10 828 696  (2 887 652) |
| 6 | 1.88 | 1 341 322  (357 686) | 3.53 | 3 801 182  (1 013 649) | 3.53 | 4 751 478  (1 267 061) | 1.18 | 1 600 626  (426 834) | 2.35 | 2 102 248  (560 599) | 13 596 856  (3 625 828) |
| 7 | 2.52 | 1 975 807  (526 882) | 4.72 | 4 752 910  (1 267 443) | 4.72 | 5 941 137  (1 584 303) | 1.57 | 1 997 179  (532 581) | 3.15 | 2 630 985  (701 596) | 17 298 018  (4 612 805) |
| 8 | 0.73 | 2 158 844  (575 692) | 1.36 | 5 027 466  (1 340 658) | 1.36 | 6 284 333  (1 675 822) | 0.45 | 2 111 578  (563 087) | 0.91 | 2 783 517  (742 271) | 18 365 738  (4 897 530) |
| 9 | 0.70 | 2 336 067  (622 951) | 1.32 | 5 293 601  (1 411 547) | 1.32 | 6 616 626  (1 764 434) | 0.44 | 2 222 342  (592 625) | 0.88 | 2 931 203  (781 654) | 19 399 538  (5 173 210) |
| 10 | 0.68 | 2 507 749  (668 733) | 1.28 | 5 550 823  (1 480 220) | 1.28 | 6 938 529  (1 850 274) | 0.43 | 2 329 643  (621 238) | 0.85 | 3 074 271  (819 806) | 20 401 015  (544 271) |
| Total | 9.95 | 11 543 454  (3 078 254) | 27.53 | 32 890 943  (8 770 918) | 27.53 | 41 113 679  (10 963 648) | 9.24 | 13 872 560  (3 699 349) | 18.30 | 18 177 546  (4 847 346) | 117 598 181  (31 359 515) |

Abbreviations: FTE, full-time equivalent; SR, Saudi Riyals; USD, United States Dollars
